# Trait anger is related to the ability to recognize facial emotions—but only in men

**DOI:** 10.3389/fpsyg.2025.1528181

**Published:** 2025-03-19

**Authors:** Anna Montag, Anette Kersting, Thomas Suslow

**Affiliations:** Department of Psychosomatic Medicine and Psychotherapy, University of Leipzig Medical Center, Leipzig, Germany

**Keywords:** trait anger, emotion recognition, facial expressions, basic emotions, anxiety, depressed mood, alexithymia

## Abstract

Trait anger is defined as a personality dimension of anger proneness. Previous research based on multimodal stimuli suggests that trait anger could be linked to poor emotion decoding. The present investigation examined the relationship between trait anger and emotion decoding ability for men and women. An emotion recognition task with images of emotional faces expressing anger, disgust, fear, sadness, surprise, or happiness was administered to 249 young adults (125 women). Participants completed the State–Trait Anger Expression Inventory (STAXI-2) along with other self-report instruments. Unbiased hit rate was calculated to assess emotion recognition accuracy. Women reported more trait anger than men. In men, but not in women, trait anger was related to negative affect variables. There were no sex differences in decoding facial emotions. For men, trait anger was negatively correlated with overall emotion recognition performance and specifically with the recognition of fear and disgust—even when controlling for relevant person variables. For women, trait anger was not related to facial emotion recognition. Compared to men with low trait anger, men with high trait anger appear to be worse at recognizing facial expressions of fear and disgust, which are negative emotions indicating being threatened or rejection.

## Introduction

Emotions are integral parts of human life. They shape our thoughts and behavior, as much as affecting our physical and mental reactions to situations, such as real time, remembered or imagined social interactions (Frijda et al., 2000; van Kleef and Côté, 2022). Furthermore, they do not only influence our decision-making (Lerner et al., 2015) but also improve our memory performance. Cahill et al. (1995) for example, have demonstrated that memory functions better if information is connected to emotion during the learning process. Experiences of basic emotions such as happiness, fear, sadness and anger can be reflected by facial expressions. Emotional facial expressions allow inferences not only about the expresser’s feeling state but also about appraisals, action requests or action tendencies (Ekman, 1993; Scherer and Grandjean, 2008). Despite their importance in social life, even at high levels of intensity, emotional facial expressions are not always correctly identified. The results of several studies on the ability to explicitly recognize and label emotions in single faces consistently indicate that facial happiness is recognized better than facial surprise, anger, disgust, and sadness whereas facial fear is identified worst among the basic emotions (Palermo and Coltheart, 2004; Goeleven et al., 2008; Kosonogov and Titova, 2019). The accuracy of facial emotion recognition appears to be linked to their frequency of occurrence in everyday life with happy faces being the most frequent expression and fear faces being the least frequent expression encountered in social contexts (Calvo et al., 2014). Studies on explicit processes of emotion recognition in single faces should be distinguished from those that deal with processes of spatial detection of emotional faces or with processes of attention allocation to facial emotions. In contrast to the result pattern of studies on the accuracy of emotion recognition in single faces, experimental findings based on visual search tasks do not indicate faster spatial detection of happy faces compared to angry faces (e.g., Horstmann and Bauland, 2006) or increased allocation of attention to happy compared to angry facial expressions at early stages of processing (Liu et al., 2021).

Anger is a commonly experienced emotion in everyday life, but the frequency of anger experiences is lower than the frequency of experience of other negative emotions such as anxiety or sadness (Trampe et al., 2015). Anger responses can be useful in social communication for reasons of self-defense (Lebel, 2017), for managing disputes and attaining personal goals (Lench et al., 2024). Important social functions of anger expression are to signal to others perceived injustice and to alter undesired outcomes by changing other persons’ behavior (Tafrate et al., 2002; Fischer and Roseman, 2007). Despite these positive functions of anger, it may have negative effects on physical health. Anger seems associated with long-term health-relevant consequences, such as earlier mortality, increased cardiovascular risk, and chronic inflammation (Harburg et al., 2003; Kerr and Schneider, 2008; Mostofsky et al., 2014; Barlow et al., 2019).

Spielberger et al. (1983) provided important impulses for a differentiated view of anger by formulating a state–trait theory of anger (Spielberger, 1988). In this approach, current experiences of anger are distinguished from habitual tendencies to experience situations as anger-provoking. The authors defined *state anger* as an emotional-physiological experience of anger, which is temporary, and related to the immediate situation, and *trait anger* as a stable tendency to experience state anger more frequently and intensely across situations (Spielberger et al., 1983, 1988, 1995; Spielberger, 1988). Thus, trait anger was viewed as a personality characteristic that is rather stable over time. Spielberger et al. (1983) assume that individuals high in trait anger tend to experience the same environmental anger triggers with more intense and enduring state anger than individuals low in trait anger. High trait anger was found to be associated with social problems such as road rage, work aggression, and domestic violence (Veenstra et al., 2018). Three main forms of anger expression can be distinguished at the dispositional level: the tendency to outwardly express anger toward other people or objects (*anger expression-out*), the tendency to direct feelings of anger inward (*anger expression-in*), and the ability to suppress angry feelings and control them by calming down when angered (*anger control*) (Spielberger, 1988; Spielberger et al., 1995). Anger is significantly interrelated with anxiety at the state and trait level in healthy and clinical samples (Mook et al., 1990; Utreja and Rizvi, 2019). State anxiety refers to unpleasant feelings of tension, apprehension, nervousness, and worry associated with activation of the autonomic nervous system, whereas trait anxiety is defined by the tendency to perceive a wide range of situations as dangerous or threatening and to respond to these threats with more frequent and intense elevations of state anxiety (Spielberger and Sydeman, 1994). Since state and trait anxiety can affect accuracy of emotion recognition in facial expressions (Surcinelli et al., 2006; Kang et al., 2019; Dyer et al., 2022), we decided to assess and control both state and trait anxiety in the present study on trait anger and facial emotion recognition.

In distinction from the emotion anger, aggression can be defined as behavior, which intends to harm other people (DeWall et al., 2012). Thus, while aggression aims at inflicting damage to another individual physically, verbally or relationally, anger is assumed to function as a preceding condition, which can initiate and energize aggressive behavior (Anderson and Bushman, 2002). Little is known about the relationship between aggression or aggressive attitudes and the ability of facial emotion recognition. Hall (2006) conducted a study to investigate the link between self-reported aggressive personality style and identification of facial anger. To this aim, she administered the facial expression receptive subtest of the Diagnostic Analysis of Nonverbal Accuracy (DANVA; Nowicki and Duke, 1994) to a sample of university students (*N* = 84). A small to medium correlation of *r* = −0.26 was found between aggressive attitude and the DANVA sum score, which reflects recognition performance across different emotion categories (i.e., happiness, sadness, anger and fear) for faces of children and adults. Thus, higher levels of dispositional aggressive tendencies (hostility, poor rage control, and the use of force as an expression of rage) seem to be linked to poor identification of emotions in other people’s faces.

There are only a few studies that have dealt with the relationship between trait anger and ability to recognize emotions in single faces. Schlegel et al. (2019) investigated the construct validity of the Geneva Emotion Recognition Test (GERT; Schlegel et al., 2014), which uses short clips with multimodal stimuli, including facial, vocal and postural, of 14 different emotional categories to assess emotion recognition. The authors observed a negative correlation of small to medium size between trait anger and the total GERT-score (*r* = −0.25) in a sample of 70 university students (40 women). According to this finding, individuals with a disposition to develop anger experiences and reactions appear characterized by a poor ability to accurately recognize other people’s emotional states from nonverbal facial, vocal, and bodily cues (Schlegel et al., 2019).

Auer et al. (2022) investigated trait anger and facial emotion recognition in essential hypertensive and normotensive men and focused on biases in emotion perception. They administered pictures with blends of two morphed basic emotions (anger to sadness, anger to fear, anger to happiness, fear to sadness, fear to happiness, and happiness to sadness) and asked participants to choose as quickly as possible which of the two possible emotions was displayed by the presented facial expression. Hypertensive men overrated anger displayed in facial expressions of mixed emotions as compared to normotensive men but there were no group differences in trait anger and trait anger did not moderate the observed group differences in anger recognition. The results of Auer et al. (2022) suggest no relationships between trait anger and recognition bias toward one emotion over others in pictures displaying mixed facial affect.

Godfrey et al. (2021) examined the relation between negative affect recognition ability and men’s past perpetration of intimate partner aggression in heterosexual couples across levels of trait anger. In this study, faces from the *Picture of Facial Affect Series* (Ekman and Friesen, 1976) expressing happiness, surprise, sadness, fear, disgust, or anger were presented to 83 men, who had to identify the displayed emotion in a forced-choice response format. The ability to recognize negative facial affect was found to be related to decreased frequency of men’s perpetration of intimate partner aggression, but only for men high in trait anger, not those low in trait anger (Godfrey et al., 2021). In the whole sample, trait anger was not correlated with negative facial affect recognition hit rate. The authors did not analyze the hit rates for single (negative) emotions.

There are a number of other psychological variables that can influence the identification of facial emotions. In recognition tasks that involve verbal labeling of expressions based on word lists participants’ verbal intelligence was found to have an impact on recognition performance (Montebarocci et al., 2011). Moreover, it has been shown that individuals suffering from depression or dysthymia are less accurate in recognizing facial expressions of emotion (Persad and Polivy, 1993; Langenecker et al., 2005; Krause et al., 2021). Alexithymia is a personality trait characterized by difficulties in identifying and verbalizing one’s emotions (Luminet et al., 2018). Alexithymia has been found to be linked to poor recognition of emotions in facial expressions (Parker et al., 1993; Mann et al., 1994). Simulation models of emotion recognition propose that at least part of the mechanism by which we identify another people’s emotions relies on internally simulating the same emotional state in ourselves (Heberlein and Atkinson, 2009). This means, when an emotional facial expression is perceived, people partially activate the respective emotion in themselves, providing a basis for the recognition of that emotion (Ross and Atkinson, 2020). Against this background it can be assumed that individuals with problems in identifying their own emotions should have also difficulties recognizing emotions in other people’s facial expressions. It can be summarized that verbal intelligence, level of depressive symptoms, and alexithymia represent psychological variables that can affect facial emotion recognition. Against this background, we decided to measure and control these factors in the present investigation.

To the best of our knowledge, the present study is the first specifically examining the association of dispositional anger and ability to recognize positive and negative emotions in faces with prototypical, unmorphed emotional facial expressions. We analyzed the relationships of trait anger with overall recognition ability and recognition performance at the level of specific emotion categories. We used unbiased hit rate, as suggested by Wagner (1993), to assess capacity of facial emotion recognition. The unbiased hit rate is insensitive to bias, number of categories, and proportions of stimuli of different types. In our emotion recognition task, seven categories of emotional facial expressions were displayed (happy, surprised, angry, disgusted, sad, fearful, and neutral expressions). Based on previous findings suggesting negative correlations of trait anger with emotion recognition ability as assessed in a task with multimodal (facial, vocal, and postural) stimuli (Schlegel et al., 2019) it was hypothesized that trait anger is associated with a reduced capacity to identify facial emotions. However, it should be noted that Godfrey et al. (2021) observed no link between trait anger and recognition of negative facial emotions in a sample of men with a history of intimate partner aggression.

We conducted separate analyses for men and women, since trait anger is linked to psychopathic traits such as fearless dominance (Edens and McDermott, 2010), which have been found to be differentially associated with recognition of negative facial emotions among men and women (Delk et al., 2017). Moreover, there is evidence that women decode facially expressed emotions better than men (Montagne et al., 2005; Thompson and Voyer, 2014) and that young, well-educated women report more dispositional anger than young, well-educated men (Rohrmann et al., 2013, p. 61).

## Materials and methods

### Participants

The current study’s sample was comprised of 125 women and 124 men with an average age of 24.27 (SD = 4.05; range: 18–35). The mean duration of participants’ school education was 12.08 years (SD = 0.69, range: 9–13). Most study participants (*n* = 186) were university students (74.7% of the sample). The other participants were in vocational training (*n* = 10), working (*n* = 44), unemployed (*n* = 8) or on parental leave (*n* = 1). Study participants were native German speakers or spoke German since the age of six. They had normal or corrected-to-normal vision as measured by standard visual acuity chart. General exclusion criteria were age under 18 and over 35 years, actual or past presence of neurological or psychiatric diseases and use of psychotropic substances (according to self-report). Participants were recruited through both social media and traditional methods (e.g., by posting recruitment ads on noticeboards). Ethical approval was obtained from the Ethics Committee at the Medical Faculty of the University of Leipzig. Informed written consent was gained from all participants.

### Questionnaires and tests

The State–Trait Anger Expression Inventory (STAXI-2; Spielberger, 1999; German version: Rohrmann et al., 2013) is a composite measure of anger consisting of five subscales (state anger, trait anger, anger expression-in, anger expression-out, and anger control). For this study, only the state and trait anger subscales were used. The state anger subscale assesses current, situational anger, whereas the trait anger subscale measures an individual’s general propensity to experience anger and its concomitant components over time.

The State–Trait Anxiety Inventory (STAI), a measure of anxious cognitive and emotional reactions, was administered in its state and trait form (German version: Laux et al., 1981). The Beck Depression Inventory (BDI-II; German version: Hautzinger et al., 2006) was used to assess participants’ level of depressive symptoms. The 20-Item Toronto Alexithymia Scale (TAS-20; German version: Bach et al., 1996) was applied to assess dispositional difficulties in identifying, describing, and attending to one’s emotions.

The Mehrfachwahl-Wortschatz-Intelligenztest (MWT-B; Lehrl, 2005), a multiple-choice vocabulary test, was administered to estimate participants’ intelligence.

### Emotion recognition experiment

Out of the Karolinska Directed Emotional Faces database (KDEF; Lundqvist et al., 1998) 140 face stimuli were selected. Each of the 10 models (five female) showed seven emotional expressions (anger, sadness, fear, disgust, surprise, happiness, and neutral) at two viewing angles (a left profile view and a frontal view). The images were in color and had a size of 14.5 cm × 14.2 cm (height and width) on the screen. In our experiment, the vertical viewing angle was 13.8° and the horizontal viewing angle was 13.5°.

Before the experimental trials, participants received 14 practice trials in which presentation conditions (seven expression qualities × two viewing angles) were displayed once. In the practice trials, images of different actors were shown (eight female and six male actors). Face stimuli were presented one at a time in the center of the screen. After a fixation cross shown for 800 ms, a face appeared on the screen for 700 ms. Then, study participants had to classify the facial expression in a forced choice manner with no time limit. They gave their answers using the number keys at the top of the keyboard: 1 (happiness), 2 (surprise), 3 (anger), 4 (disgust), 5 (anxiety), 6, (sadness), and 7 (neutral). The arrangement of the response keys was the same for all participants and did not change during the experiment. The emotional expression categories and the assigned numbers were shown after the face stimulus in white letters at the bottom of a black screen until a response was made. The interval between trials was 2 s.

Before the experiment, participants were told that they would see faces expressing happiness, surprise, anger, disgust, anxiety, or sadness and faces with a neutral expression and that some faces would be seen from the side, others in frontal view. Participants had the task of identifying the expression of each face and responding as accurately as possible. No feedback on the correctness of responses was given. After 50 and 100 trials, participants had a short break. The breaks each lasted about a minute. Experimental trials were presented in a fixed random order with the constraints that not more than three subsequent trials show the same emotion category and that no two subsequent trials depict the same actor. During the task, participants sat in a chair at approximately 60 cm in front of a 15.6-inch screen of a Dell Latitude E6510. The software *Inquisit* 3.0 (Draine, 2004) was used for stimulus presentation and response registration.

### General procedure

The experimental sessions were conducted individually at the Department of Psychosomatic Medicine and Psychotherapy, University of Leipzig. All study participants were tested in a quiet room. Questionnaires and tests were administered in a fixed sequence: STAI-State, TAS-20, STAXI-2, STAI-Trait, MWT-B, and the BDI-II. Finally, participants performed the emotion recognition experiment.

### Statistical analysis

All 249 study participants had complete data sets. Independent *t*-tests were computed to examine differences between women and men in sociodemographic and psychological characteristics. Accuracy of facial emotion recognition was assessed using Wagner’s (1993) unbiased hit rate (H_u_). The unbiased hit rate varies between 0 and 1 expressing identification accuracy as proportions of both stimulus and response frequency. Unbiased hit rates were calculated for each participant as the squared frequency of correct responses for a target emotion divided by the product of the overall frequency that this emotion category is chosen and the number of stimuli representing this emotion. For each participant, frequency values were pooled across viewing angle conditions. Unbiased hit rates were computed for the seven facial expression conditions. In addition, an overall unbiased hit rate was calculated for the recognition task. The overall hit rate was formed by adding up the seven individual hit rates and dividing by the number of facial expression conditions. Emotion identification data were analyzed by a mixed model ANOVA with the repeated measure factor emotional expression (happiness, surprise, anger, disgust, fear, sadness, and neutrality) and the between-subject factor biological sex (woman, man). Greenhouse–Geisser correction was applied to correct degrees of freedom of F-ratios in case the sphericity assumption was violated (Greenhouse and Geisser, 1959). To analyze pairwise differences in hit rates between emotion conditions we performed Bonferroni-adjusted pairwise comparisons.

Product moment correlation was used to examine the relationships between state and trait anger, state and trait anxiety, depression, alexithymia, sociodemographic variables, intelligence, and accuracy of facial emotion identification in the female and male sample. In addition, hierarchical regression analyses were performed for those unbiased hit rates, which showed correlations with trait anger, to examine whether these relationships remain significant after adjusting the effects of other relevant variables. Results were considered significant at *p* < 0.05, two-tailed. Statistical analyses were made with SPSS software version 29.0 (IBM Corp., Armonk, NY, USA).

An *a priori* analysis of statistical power was computed with the program G*Power (version 3.1.9.2.; bivariate normal model—exact test family) of Faul et al. (2009). To detect a small to medium effect of *r* = 0.25 (cf. Schlegel et al., 2019) with an alpha value of 0.05, two-tailed, and a power of 0.80 the required total sample size is 123.

## Results

### Sociodemographic and psychological variables: comparison between women and men

Descriptive statistics of sociodemographic data and psychological characteristics for women and men are presented in Table 1. In our study, men were significantly older than women (on average about one and a half year). Women had higher trait anger scores, and higher trait anxiety scores compared to men (see Table 1 for details). There were no differences between the sexes regarding years of school education, intelligence, state anger, state anxiety, level of depressive symptoms, and alexithymia. Note that 65% of our male participants and 72% of our female participants reported to have felt no state anger at all. Because of the skewed distributions of the state anger scores, we used Spearman rank coefficients in the following correlation analyses. Comparing the state anger scores of men and women on the basis of the Mann–Whitney-*U*-test yielded a nonsignificant result similar to that of the *t*-test reported in Table 1 (*Z* = −1.45, *p* = 0.15).

**Table 1:** Sociodemographic and psychological test data for women and men (means and SDs (in parentheses)).

Variable	Women (*n* = 125)	Men (*n* = 124)	*t*	*p*
Age (years)	23.54 (4.00)	25.00 (4.00)	−2.89	0.004**
School education (years)	12.10 (0.68)	12.06 (0.71)	0.45	0.654
Intelligence (MWT-B, IQ)	110.66 (10.04)	110.26 (11.91)	0.29	0.771
State anger (STAXI-2)	15.82 (1.93)	16.31 (2.47)	−1.72	0.087
Trait anger (STAXI-2)	20.54 (4.44)	18.61 (4.18)	3.53	<0.001***
State anxiety (STAI)	35.24 (6.84)	35.20 (6.31)	0.05	0.963
Trait anxiety (STAI)	40.57 (8.47)	38.06 (9.00)	2.27	0.024*
Depressivity (BDI-II)	8.74 (6.02)	8.11 (6.72)	0.77	0.441
Alexithymia (TAS-20)	41.32 (9.53)	41.77 (9.77)	−0.37	0.711

*p < 0.05; **p < 0.01; ***p < 0.001 (two-tailed). MWT-B, IQ, Multiple-choice vocabulary test version B, intelligence quotient; STAXI-2, State–Trait Anger Expression Inventory - 2; STAI, State–Trait Anxiety Inventory; BDI-II, Beck Depression Inventory II; TAS-20, 20-Item Toronto-Alexithymia Scale.

### Relationships of trait anger with sociodemographic and psychological variables in the female and male sample

In our female sample, trait anger was positively correlated with age and alexithymia. For women, no correlations were observed between trait anger and school education, intelligence, and scales assessing negative affectivity (see Table 2). In contrast, in the male sample we found a negative correlation of trait anger with school education and positive correlations of trait anger with state anger, state anxiety, and trait anxiety. Moreover, trait anger was also positively correlated with alexithymia (see Table 2 for details).

**Table 2:** Correlations of trait anger (STAXI-2) scales with age, school education, intelligence (MWT-B), state anger (STAXI-2), state and trait anxiety (STAI), depressivity (BDI-II), and alexithymia (TAS-20) as a function of sex.

Variable	Women (*n* = 125)	Men (*n* = 124)
	Trait anger	Trait anger
Age	0.23*	−0.07
School education	0.07	−0.21*
Intelligence	−0.01	0.00
State anger^#^	0.02	0.32***
State anxiety	0.03	0.24**
Trait anxiety	0.20	0.28**
Depressivity	0.08	0.14
Alexithymia	0.31***	0.26**

*p ≤ 0.05; **p ≤ 0.01; ***p ≤ 0.001 (two-tailed). ^#^Spearman rank correlations.

### Facial emotion recognition: comparison between women and men

Mean unbiased hit rates as a function of emotional category of facial expression and sex are presented in Table 3. A 7 × 2 mixed ANOVA on hit rates yielded only a main effect of emotional category of facial expression [*F*_(4.58, 1130.21)_ = 624.76, *p* < 0.001, $\eta_{p}^{2}$ = 0.72]. No other significant effects were observed (all *p*s > 0.23). That means, women did not differ from men in the ability to identify facial emotions. According to Bonferroni-adjusted pairwise comparisons, happiness (M = 0.908, SD = 0.005) was recognized better than neutral expression (M = 0.774, SD = 0.010), neutral expression better than anger (M = 0.732, SD = 0.010), anger better than disgust (M = 0.689, SD = 0.009), disgust better than sadness (M = 0.628, SD = 0.009) and surprise (M = 0.623, SD = 0.006), and sadness and surprise (which did not differ) were recognized better than fear (M = 0.343, SD = 0.011) (all *p*s < 0.001). Thus, recognition performance was best for happiness and worst for fear.

**Table 3:** Correlations of state and trait anger (STAXI-2) with unbiased hit rates for facial expressions in the emotion recognition task for women and men [with descriptive statistics (means and SDs) for hit rates].

	Overall hit rate	Anger	Fear	Disgust	Sadness	Surprise	Happiness	Neutral
Women (*n* = 125)								
State anger^#^	**−0.17**	−0.12	−0.08	−0.07	−0.13	0.01	−0.04	−0.15
Trait anger	**0.02**	−0.03	−0.04	−0.08	0.12	0.06	−0.04	0.11
Mean	***0.68***	*0.74*	*0.35*	*0.69*	*0.65*	*0.63*	*0.91*	*0.77*
SD	***0.09***	*0.14*	*0.17*	*0.14*	*0.14*	*0.09*	*0.07*	*0.16*
Men (*n* = 124)								
State anger^#^	**−0.13**	−0.21*	−0.08	−0.07	−0.02	−0.22*	0.07	−0.08
Trait anger	−0.20*	−0.17	−0.23**	−0.20*	−0.19*	−0.13	0.05	−0.03
Mean	***0.66***	*0.73*	*0.33*	*0.69*	*0.61*	*0.61*	*0.90*	*0.78*
SD	***0.10***	*0.17*	*0.16*	*0.14*	*0.16*	*0.10*	*0.09*	*0.14*

*p ≤ 0.05; **p ≤ 0.01 (two-tailed). ^#^Spearman rank correlations. Correlations of overall hit rate are printed in bold. Means and SDs are printed in italics.

To explore the effect of viewing angle in our experiment we calculated a 7 (emotional category) × 2 (viewing angle) × 2 (biological sex) ANOVA on hit rates (unbiased hit rates as a function of emotional category of facial expression, viewing angle, and sex are presented in Supplementary Table 1). There were significant main effects of emotional category of facial expression [*F*_(4.55, 1123.62)_ = 613.38, *p* < 0.001, $\eta_{p}^{2}$ = 0.71], and viewing angle [*F*_(1, 247)_ = 78.54, *p* < 0.001, $\eta_{p}^{2}$ = 0.24], and a significant interaction between emotional category and viewing angle [*F*_(4.71, 1164.53)_ = 43.17, *p* < 0.001, $\eta_{p}^{2}$ = 0.15]. No other significant effects were found (all *p*s ≥ 0.20). Thus, women did not differ from men in the ability to identify emotions in profile and frontal views of facial expressions. Results from Bonferroni-adjusted pairwise comparisons indicated that happiness (M = 0.950, SD = 0.078 vs. M = 0.869, SD = 0.109), surprise (M = 0.699, SD = 0.130 vs. M = 0.561, SD = 0.110), fear (M = 0.413, SD = 0.208 vs. M = 0.291, SD = 0.172), and neutral expressions (M = 0.794, SD = 0.198 vs. M = 0.764, SD = 0.163) were recognized significantly better in frontal than in profile view (all *p*s < 0.05). For angry (M = 0.737, SD = 0.162 vs. M = 0.737, SD = 0.187) and sad facial expressions (M = 0.644, SD = 0.159 vs. M = 0.625, SD = 0.196) no differences in hit rates were observed. Disgusted faces were identified better in profile than in frontal view (M = 0.721, SD = 0.174 vs. M = 0.669, SD = 0.162) (*p* < 0.001).

### Relationships of trait anger with facial emotion recognition in the female and male sample

For women, neither trait anger nor state anger was correlated with hit rates in the emotion recognition task (see Table 3). In the male sample, trait anger was negatively correlated with overall hit rate in the emotion recognition task. Analyses at the level of specific emotional expressions showed that trait anger was negatively correlated with hit rates for fear, disgust, and sadness, while state anger was negatively correlated with hit rate for anger and surprise (see Table 3).

A regression model for overall hit rate was computed to investigate whether trait anger is a predictor independent from state anger, state anxiety, trait anxiety, alexithymia, and school education. In the first step of the hierarchical regression analysis, variance in overall hit rate was significantly explained by state anger and state anxiety, with individuals with higher values showing worse recognition (see Table 4). In step two entering trait anger, significantly increased the predictive value of the model. This means, trait anger was found to be a negative predictor of the overall hit rate in the emotion recognition experiment (see Table 4).

**Table 4:** Hierarchical regression predicting the overall unbiased hit rate for facial expressions in the emotion recognition task in two steps by school education, state anger (STAXI-2), state anxiety (STAI), trait anxiety (STAI), and alexithymia (TAS-20), and trait anger (STAXI-2) in the male sample (*n* = 124).

Predictor	Coefficients				Multicollinearity		Model	
	*β*	Beta	*t*	Sig. (*p*)	Tol.	VIF	R^2^	∆R^2^
Step 1								
State anger	−0.008	−0.207	−2.09	0.038*	0.78	1.28	0.099	–
State anxiety	−0.004	−0.240	−2.23	0.027*	0.66	1.51		
Trait anxiety	0.002	0.209	1.82	0.071	0.58	1.73		
Alexithymia	0.001	0.136	1.27	0.207	0.66	1.51		
School education	0.009	0.063	0.68	0.496	0.91	1.10		
Step 2								
State anger	−0.006	−0.160	−1.60	0.113	0.74	1.35	0.131	0.032*
State anxiety	−0.004	−0.230	−2.17	0.032*	0.66	1.51		
Trait anxiety	0.002	0.231	2.03	0.044*	0.57	1.75		
Alexithymia	0.001	0.147	1.39	0.168	0.66	1.51		
School education	0.004	0.030	0.33	0.742	0.88	1.13		
Trait anger	−0.005	−0.196	−2.08	0.040*	0.83	1.20		

*β*, unstandardized regression coefficient; Tol., Tolerance; VIF, Variance Inflation Factor. *p ≤ 0.05 (two-tailed).

We calculated additional regression models concerning hit rates for the specific emotions fear, disgust, and sadness entering trait anger in the second step as predictor. Trait anger was a significant negative predictor of hit rate for fear (see Supplementary Table 2) and hit rate for disgust (see Supplementary Table 3). A further regression analysis showed that trait anger did not significantly predict hit rate for sadness after adjusting the effects of state anger, state anxiety, trait anxiety, alexithymia, and school education (see Supplementary Table 4).

Additional correlation analyses were carried out to explore the relationships of trait anger with emotion recognition in faces shown in frontal and in profile view for men and women separately. In the female sample, no significant correlations were observed between trait anger and emotion recognition in faces shown in frontal or in profile view. In the male sample, trait anger showed significant negative correlations with hit rates for fear in faces presented in frontal and in faces presented in profile view, for disgust in faces presented in profile view, and for sadness in faces presented in frontal view (see Supplementary Table 5 for details). According to Steiger’s *Z* the correlation between trait anger and hit rate for disgust in frontally presented faces was not lower than the correlation between trait anger and hit rate for disgust in profile faces (*Z* = 0.62, *p* = 0.27). Moreover, the correlation between trait anger and hit rate for sadness in profile faces was not lower than the correlation between trait anger and hit rate for sadness in frontally presented faces (*Z* = −0.73, *p* = 0.23).

## Discussion

In this study, we investigated the relationship between dispositional anger and the ability to recognize emotions in single faces. Correct understanding of facial emotions seems highly relevant for smooth and efficient social interaction (Ferretti and Papaleo, 2019; Kroczek et al., 2024). High trait anger is known to be linked to interpersonal problems and social maladjustment (Birkley and Eckhardt, 2015; Veenstra et al., 2018). We computed unbiased hit rates and analyzed emotion recognition accuracy separately for men and women. There are indications that, in general, women decode facial emotions better than men (Montagne et al., 2005; Thompson and Voyer, 2014), and that well-educated women report more dispositional anger than well-educated men (Rohrmann et al., 2013). In the present study, we found no evidence for sex differences in facial emotion recognition (neither for frontally presented faces nor for faces shown in profile), but, consistent with previous data, heightened dispositional anger in women compared to men. The reasons for this have not been clarified. Heightened trait anger in women could be related, for example, to a greater propensity to express emotions (Kring and Gordon, 1998). Martin et al. (2000) observed that women scored higher than men on the affective component of trait anger but not on the behavioral and cognitive components. General disadvantages women face concerning the access to economic and social resources could be other possible reasons for heightened trait anger (Thomas, 1993; Burchi and Malerba, 2023). However, not consistent with this assumption, we found no association between trait anger and education in our female participants.

Interestingly, although women reported more trait anger (and more trait anxiety) than men trait anger in women was not related to other negative affect variables. In contrast, in the male sample trait anger was positively correlated with state anger, state anxiety, and trait anxiety. This means that in men the inclination to feel anger in everyday life is associated with the tendency to perceive the environment as threatening, experience helplessness and lack of control. In our sample, the mean trait anger scores of women and men (20.5 and 18.6 respectively) were within the average range [percentile ranks 47 (for women) and 45 (for men)] compared to German norms (for the age span 16–39 years; Rohrmann et al., 2013). Our emotion recognition data basically confirm previous findings showing that, among the basic emotions, facial happiness is recognized best, whereas facial fear is identified worst (cf. Palermo and Coltheart, 2004; Goeleven et al., 2008; Kosonogov and Titova, 2019). In our study, we found differences in facial emotion recognition as a function of view. Happy, fearful, surprised, and neutral faces were identified better in frontal than in profile view. In contrast, facial disgust was recognized better in profile than in frontal view. For angry and sad facial expressions there were no differences in hit rates. Our findings are only in part consistent with previous research findings. Surcinelli et al. (2022) found better recognition of facial fear, sadness, and anger in frontal compared to profile view. Guo and Shaw (2015) observed better recognition of facial disgust and sadness in frontal than in profile view. In contrast, Matsumoto and Hwang (2011) reported no differences in emotion recognition between faces in frontal view and those in profile view. All in all, the findings so far on the effect of viewing angle on facial emotion recognition are rather heterogeneous and could indicate that the pattern of results might depend on the face database administered in the studies or the specific faces selected from the face databases.

The results of the present investigation corroborate our assumption that dispositional anger is associated with a reduced capacity to decode facial emotion for men. This assumption was not confirmed for women. Thus, our hypothesis was only partially supported. According to our data, in the male sample trait anger was negatively related to overall emotion recognition performance independently from state anger, state and trait anxiety, alexithymia, and school education. Further analyses at the level of specific emotional expressions revealed that trait anger was negatively associated with the recognition of facial fear, disgust, and sadness. After controlling other relevant variables trait anger predicted hit rate for fear and disgust but not hit rate for sadness. The observed correlations had a small to medium effect size. The present results partially confirm and specify the findings of Schlegel et al. (2019) in a mixed sample of university students indicating a negative correlation between trait anger and emotion recognition in nonverbal multimodal stimuli. Our data is also in line with the observation by Hall (2006) that an aggressive personality style is associated with poor identification of emotions in other people’s faces.

Additional analyses of our data in the male sample, which differentiated between facial emotion recognition in frontal and profile views, showed that trait anger was significantly associated with fear recognition in faces displayed in frontal view as well as in faces presented in profile, with disgust recognition only in faces shown in profile, and with sadness recognition only in faces displayed in frontal view. This correlation pattern suggests that some of the associations between dispositional anger and facial emotion recognition might depend on viewing angle. Thus, it appears that for some emotion categories (such as disgust) perceptual factors may have an impact on the relationship between dispositional anger and emotion recognition. It is assumed that perceptual, semantic, and affective information is extracted from emotional facial expressions, and together they contribute to emotion recognition (Calvo and Nummenmaa, 2016). In particular, expression recognition in explicit emotion categorization tasks is thought to rely strongly on perceptual processes. Against this background, future research on dispositional anger and facial emotion recognition should consider presenting faces in different views and try to specify which of the perceptual, affective, and semantic processes involved in expression recognition are less efficient in men with high trait anger compared to those low in trait anger.

Our findings are not consistent with those of Auer et al. (2022) who observed no relationship between trait anger and recognition bias toward one emotion over others in pictures displaying mixed facial affect. The differences in results could be due to specific characteristics of Auer et al. (2022) study: the authors investigated men who were about 50 years old and administered morphed pictures of facial affect—our male sample had instead an average age of only 25 years and in our investigation unmorphed pictures of basic emotions were presented. Moreover, half of the sample of Auer et al. (2022) suffered from essential hypertension whereas our sample consisted of healthy men. Finally, participants in Auer et al. (2022) study had to make decisions as quickly as possible whereas in our study participants classified facial expressions without time limit. The findings of our study are also not in line with those of Godfrey et al. (2021) who found no evidence in their total sample for a correlation of trait anger with negative facial affect recognition. However, Godfrey et al. (2021) examined a specific sample of men: the large majority of their male participants had manifested male-to-female intimate partner aggression. The authors observed that trait anger moderated the relation between frequency of men’s perpetration of intimate partner aggression and recognition of negative facial affect. Only for men high in trait anger, the ability to identify negative facial affect was associated with decreased frequency of men’s perpetration of partner aggression.

For men, we found associations of state anger with recognition of facial anger and surprise. However, the latter finding must be interpreted cautiously due to the very low state anger scores in our sample. In contrast to the male sample, there were no correlations of trait and state anger with emotion recognition in the female sample. It seems that in women dispositional anger and ability to decode emotions in faces are independent from each other.

Because negative emotions expressed by others can inhibit anger reactions and aggressive behavioral tendencies, poor recognition of others’ negative emotions might maintain feelings of anger and perpetuate or even escalate interpersonal conflict and tension in men with high trait anger. Facial expressions of disgust indicate a request for greater distance (Horstmann, 2003) and interpersonal rejection (Sherman and Haidt, 2011). If people become aware that they may cause fear and worries or wishes to increase distance in others they should rather control or downregulate their anger reactions. According to the Violence Inhibition Mechanism (VIM) model (Blair et al., 2004) healthy individuals avoid behaviors, which result in distressing feelings in others, especially fear and sadness. In this theoretical context, it has been argued that people with difficulties in identifying distressing feelings in others should be more inclined to engage in aggression due to the lack of initiation of violence inhibition compared to people with good recognition abilities (Blair, 1995). Godfrey et al. (2021) point out that deficiencies in recognition of facial emotions could play a significant role in the maintenance of aggressive behaviors. Future longitudinal research has to clarify whether improvements in facial affect recognition through training can have positive effects on social functioning and emotional wellbeing in individuals with high trait anger—or in the long term even reduce their level of trait anger.

The present study has several limitations. Our study participants were young, well-educated individuals, which clearly limits the generalizability of our results. Moreover, the facial expressions displayed in our experiment were static images showing emotions at high intensities. It can be criticized that in everyday life emotional facial expressions are in general dynamic and emotions are frequently expressed at low intensity levels. Furthermore, we used only self-report to assess participants’ anger experience in our study. Future research may incorporate also objective measures of anger reactivity during anger-eliciting situations (Potegal and Qiu, 2010).

To sum up, men, but not women, with a disposition to develop anger experiences and reactions appear characterized by a poor ability to recognize fear and disgust in other people’s facial expressions. The ability to recognize negative emotions in others should make the impact of own behavior on others more accessible. Our results may help to better understand anger-related interpersonal problems in men. In our study, men did not differ in facial emotion recognition from women, and recognition performance was best for happiness and worst for fear.

## Data Availability

The raw data supporting the conclusions of this article will be made available by the authors, without undue reservation.
