# Supplementary material for: Trait anger is related to the ability to recognize facial emotions—but only in men
Source: Front Psychol. 2025 Mar 19;16:1528181. doi: 10.3389/fpsyg.2025.1528181 (PMC11962005; doi:10.3389/fpsyg.2025.1528181)
Supplement: Supplementary file 1 [file Table_1.DOCX]

Supplementary Table 1: Unbiased hit rates for facial expressions as a function of viewing angle in the emotion recognition task for women and men (means and SDs).

| **Women:** | Anger | Fear | Disgust | Sadness | Surprise | Happiness | Neutral |
| --- | --- | --- | --- | --- | --- | --- | --- |
| **Frontal** Mean  view *SD* | 0.74 | 0.42 | 0.67 | 0.67 | 0.71 | 0.96 | 0.79 |
|  | *0.15* | *0.21* | *0.16* | *0.14* | *0.13* | *0.07* | *0.20* |
| **Profile** Mean  view *SD* | 0.74 | 0.30 | 0.71 | 0.65 | 0.57 | 0.88 | 0.77 |
|  | *0.18* | *0.18* | *0.18* | *0.19* | *0.11* | *0.11* | *0.16* |
| **Men:** | Anger | Fear | Disgust | Sadness | Surprise | Happiness | Neutral |
| **Frontal** Mean  view *SD* | 0.73 | 0.40 | 0.67 | 0.62 | 0.69 | 0.94 | 0.80 |
|  | *0.17* | *0.21* | *0.16* | *0.17* | *0.13* | *0.09* | *0.19* |
| **Profile** Mean  view *SD* | 0.73 | 0.28 | 0.73 | 0.60 | 0.55 | 0.86 | 0.76 |
|  | *0.20* | *0.16* | *0.17* | *0.20* | *0.11* | *0.11* | *0.16* |
