# Supplementary material for: Trait anger is related to the ability to recognize facial emotions—but only in men
Source: Front Psychol. 2025 Mar 19;16:1528181. doi: 10.3389/fpsyg.2025.1528181 (PMC11962005; doi:10.3389/fpsyg.2025.1528181)
Supplement: Supplementary file 2 [file Table_2.DOCX]

Supplementary Table 2: Hierarchical regression predicting the unbiased hit rate for facial fear in the emotion recognition task in two steps by school education, state anger (STAXI-2), state anxiety (STAI), trait anxiety (STAI), and alexithymia (TAS-20), and trait anger (STAXI-2) in the male sample (n = 124).

|  | **Coefficients Multicollinearity Model** | | | | | | | |
| --- | --- | --- | --- | --- | --- | --- | --- | --- |
| **Predictor** | **β** | **Beta** | ***t*** | **Sig. (*p*)** | **Tol.** | **VIF** | **R^2^** | ∆**R^2^** |
| **Step1** State anger | -.009 | -.132 | -1.29 | .200 | .78 | 1.28 | .032 | - |
| State anxiety | .000 | -.012 | -0.10 | .917 | .66 | 1.51 |  |  |
| Trait anxiety | .001 | .033 | 0.27 | .784 | .58 | 1.73 |  |  |
| Alexithymia | .003 | .175 | 1.57 | .120 | .66 | 1.51 |  |  |
| School  education | .008 | .035 | 0.36 | .715 | .91 | 1.10 |  |  |
| **Step2** State anger | -.004 | -.064 | -0.63 | .530 | .74 | 1.35 | .098 | .066** |
| State anxiety | .000 | .002 | 0.02 | .982 | .66 | 1.51 |  |  |
| Trait anxiety | .001 | .064 | 0.56 | .579 | .57 | 1.75 |  |  |
| Alexithymia | .003 | .190 | 1.76 | .081 | .66 | 1.51 |  |  |
| School  education | -.003 | -.012 | -0.12 | .901 | .88 | 1.13 |  |  |
| Trait anger | -.011 | -.281 | -2.92 | .004** | .83 | 1.20 |  |  |

β = unstandardized regression coefficient, Tol. = Tolerance, VIF = Variance Inflation Factor

* *p* ≤ 0.05, ** *p* ≤ 0.01 (two-tailed).
