# Supplementary material for: Trait anger is related to the ability to recognize facial emotions—but only in men
Source: Front Psychol. 2025 Mar 19;16:1528181. doi: 10.3389/fpsyg.2025.1528181 (PMC11962005; doi:10.3389/fpsyg.2025.1528181)
Supplement: Supplementary file 3 [file Table_3.DOCX]

Supplementary Table 3: Hierarchical regression predicting the unbiased hit rate for facial disgust in the emotion recognition task in two steps by school education, state anger (STAXI-2), state anxiety (STAI), trait anxiety (STAI), and alexithymia (TAS-20), and trait anger (STAXI-2) in the male sample (n = 124).

|  | **Coefficients Multicollinearity Model** | | | | | | | |
| --- | --- | --- | --- | --- | --- | --- | --- | --- |
| **Predictor** | **β** | **Beta** | ***t*** | **Sig. (*p*)** | **Tol.** | **VIF** | **R^2^** | ∆**R^2^** |
| **Step1** State anger | -.009 | -.167 | -1.67 | .098 | .78 | 1.28 | .077 | - |
| State anxiety | -.005 | -.228 | -2.10 | .038* | .66 | 1.51 |  |  |
| Trait anxiety | .003 | .183 | 1.57 | .118 | .58 | 1.73 |  |  |
| Alexithymia | .002 | .120 | 1.10 | .274 | .66 | 1.51 |  |  |
| School  education | .010 | .050 | 0.54 | .589 | .91 | 1.10 |  |  |
| **Step2** State anger | -.007 | -.120 | -1.18 | .240 | .74 | 1.35 | .109 | .032* |
| State anxiety | -.005 | -.218 | -2.03 | .044* | .66 | 1.51 |  |  |
| Trait anxiety | .003 | .205 | 1.78 | .078 | .57 | 1.75 |  |  |
| Alexithymia | .002 | .131 | 1.22 | .227 | .66 | 1.51 |  |  |
| School  education | .004 | .018 | 0.19 | .847 | .88 | 1.13 |  |  |
| Trait anger | -.007 | -.196 | -2.04 | .043* | .83 | 1.20 |  |  |

β = unstandardized regression coefficient, Tol. = Tolerance, VIF = Variance Inflation Factor

* *p* ≤ 0.05 (two-tailed).
