# Supplementary material for: Trait anger is related to the ability to recognize facial emotions—but only in men
Source: Front Psychol. 2025 Mar 19;16:1528181. doi: 10.3389/fpsyg.2025.1528181 (PMC11962005; doi:10.3389/fpsyg.2025.1528181)
Supplement: Supplementary file 4 [file Table_4.DOCX]

Supplementary Table 4: Hierarchical regression predicting the unbiased hit rate for facial sadness in the emotion recognition task in two steps by school education, state anger (STAXI-2), state anxiety (STAI), trait anxiety (STAI), and alexithymia (TAS-20), and trait anger (STAXI-2) in the male sample (n = 124).

|  | **Coefficients Multicollinearity Model** | | | | | | | |
| --- | --- | --- | --- | --- | --- | --- | --- | --- |
| **Predictor** | **β** | **Beta** | ***t*** | **Sig. (*p*)** | **Tol.** | **VIF** | **R^2^** | ∆**R^2^** |
| **Step1** State anger | -.007 | -.115 | -1.13 | .259 | .78 | 1.28 | .046 | - |
| State anxiety | -.004 | -.164 | -1.48 | .141 | .66 | 1.51 |  |  |
| Trait anxiety | .003 | .177 | 1.49 | .138 | .58 | 1.73 |  |  |
| Alexithymia | .001 | .056 | 0.51 | .612 | .66 | 1.51 |  |  |
| School  education | .023 | .103 | 1.10 | .275 | .91 | 1.10 |  |  |
| **Step2** State anger | -.004 | -.070 | -0.68 | .499 | .74 | 1.35 | .076 | .030 |
| State anxiety | -.004 | -.154 | -1.41 | .161 | .66 | 1.51 |  |  |
| Trait anxiety | .003 | .198 | 1.69 | .095 | .57 | 1.75 |  |  |
| Alexithymia | .001 | .067 | 0.61 | .543 | .66 | 1.51 |  |  |
| School  education | .016 | .072 | 0.76 | .446 | .88 | 1.13 |  |  |
| Trait anger | -.007 | -.189 | -1.93 | .055 | .83 | 1.20 |  |  |

β = unstandardized regression coefficient, Tol. = Tolerance, VIF = Variance Inflation Factor
