# Supplementary material for: Trait anger is related to the ability to recognize facial emotions—but only in men
Source: Front Psychol. 2025 Mar 19;16:1528181. doi: 10.3389/fpsyg.2025.1528181 (PMC11962005; doi:10.3389/fpsyg.2025.1528181)
Supplement: Supplementary file 5 [file Table_5.DOCX]

Supplementary Table 5: Correlations of trait anger (STAXI-2) with unbiased hit rates for facial expressions as a function of viewing angle in the emotion recognition task for men (n = 124).

|  | Anger | Fear | Disgust | Sadness | Surprise | Happiness | Neutral |
| --- | --- | --- | --- | --- | --- | --- | --- |
| Frontal view | -.16 | -.21* | -.14 | -.20* | -.07 | .07 | -.10 |
| Profile view | -.15 | -.21* | -.20* | -.13 | -.15 | .03 | .05 |

* *p* ≤ 0.05
